# Casein Microgels as Benzydamine Hydrochloride Carriers for Prolonged Release

**DOI:** 10.3390/ma15041333

**Published:** 2022-02-11

**Authors:** Sofia Milenkova, Bissera Pilicheva, Yordanka Uzunova, Temenuzhka Yovcheva, Maria Marudova

**Affiliations:** 1 Department of Physics, Faculty of Physics and Technology, University of Plovdiv “Paisii Hilendarski”, 24 Tzar Asen Str., 4000 Plovdiv, Bulgaria; sophiamilenkowa@gmail.com (S.M.); temiov@uni-plovdiv.bg (T.Y.); 2 Faculty of pharmacy, Medical University of Plovdiv, 15A Vassil Aprilov Blvd, 4002 Plovdiv, Bulgaria; bisera.pilicheva@mu-plovdiv.bg (B.P.); yordanka.uzunova@mu-plovdiv.bg (Y.U.); 3 Research Institute, Medical University of Plovdiv, 15A Vassil Aprilov Blvd, 4002 Plovdiv, Bulgaria

**Keywords:** benzydamine, casein, crosslinking, drug delivery

## Abstract

This research aims to investigate the properties of nano- and micro-sized casein hydrogels crosslinked by sodium tripolyphosphate as drug delivery systems. Benzydamine hydrochloride was chosen as a model hydrophilic drug. The gels were synthesized by varying different parameters: casein concentration, casein/crosslinking ratio, and addition of ethanol as a co-solvent. The electrostatic attractive interactions between the casein and the sodium tripolyphosphate were confirmed by FTIR spectroscopy. The particle sizes was determined by dynamic light scattering and varied in the range between several hundred nanometers and several microns. The yield of the gelation process was high for all investigated samples and varied between 55.3% and 78.3%. The encapsulation efficiency of the particles was strongly influenced by the casein concentration and casein/crosslinker ratio and its values were between 4.6% and 22.4%. The release study confirmed that casein particles are useful as benzydamine carriers and ensured prolonged release over 72 h.

## 1. Introduction

Recently, naturally based micro- and nano-structures, and especially protein particles, have been widely used in the pharmaceutical and medical fields due to a couple of benefits. Firstly, these particles inherit the low toxicity and biodegradability of the proteins [1]. Secondly, proteins exhibit unique properties and functional abilities (including gelation, emulsification, water binding capacity, etc.). For example, their structures contain different functional groups, enabling interactions between the protein and a wide range of active compounds. Moreover, these interactions are reversible, meaning that under certain conditions, these compounds could be released out of the system [2]. Protein-based particles also show other important advantages in their potential use as a drug delivery system, such as stability, surface modification possibility, and control over their size [3,4]. Furthermore, they can improve the stability of the encapsulated agent, and ensure its longer half-life by protecting it from enzymatic degradation and/or renal clearance [5]. These factors are attracting the attention toward particles on the base of proteins and their application for cancer therapy [6], lung-targeted delivery [7], and even vaccines, due to their non-antigenic properties [8].

Casein (CAS) is the major protein in milk and dairy products, and it is an essential part of nutrition worldwide. It is biodegradable, biocompatible, and nontoxic. This complex of beneficial properties makes it desirable in the development of drug delivery systems.

The chemical structure of casein is characterized by a variety of functional groups such as carboxylic, amines, and phosphates. Depending on the pH conditions, these groups create opportunities for crosslinking and network formation and stabilization [9], polymer conjugation [10,11], or drug binding via chemical [12], hydrophobic [13], or ionic interactions [14].

Casein is composed of 94% protein and 6% low molecular weight compounds. Four types of casein phosphoproteins are found in cow milk, namely αS1-, αS2-, β-, and κ-casein, which exist in the approximate weight proportions of 4:1:4:1 [15]. When it is in aqueous solution, CAS forms stable micelles. On the base of these micelles, a wide variety of structures can be obtained by applying different techniques or technical approaches. The most commonly used structures are micro- and nano-particles, hydrogels, and other composites as vesicles for biologically active compounds [16]. Casein micelles are formulated on the base of the aforementioned phosphoproteins. According to the hypothesis and experimental work of Gandhi et al. [17], the addition of excess amount of Ca^2+^ leads to a more packed structure and conjugation of more free casein molecules into the micelles. These micelles can be held together by two main factors: hydrophobic interactions and calcium phosphate nanoclusters. The second one plays a vital role in the integrity of the micellar structures. In the Holt model examining the micelle structure, each micelle is a colloidal particle consisting of about a thousand smaller nanoclusters. These nanoclusters are playing the role of building blocks of the self-assembled micelle structure [18]. The outer surface of the micelles is mainly constructed of κ-casein phosphoprotein, ensuring hydrophilicity and surface charge, helping their stability through inter-micellar electrostatic and steric repulsion [19]. The behavior of micelles is highly dependent on pH. They swell at higher pH values due to increased electrostatic repulsions, leading to loosening of the micellar structure [20]. At neutral pH, casein micelles behave as hard spheres, based on their rheological and diffusion behavior [21]. A precipitation process can be observed around pH = 4.6, which is the isoelectric point for casein [22]. Crosslinked micelles exhibit higher stability in comparison to non-crosslinked micelles [23].

Benzydamine hydrochloride (Benz) is a well-known non-steroidal anti-inflammatory drug with an antimicrobial effect, along with analgesic and anesthetic ones [24]. It can be applied both locally and systemically, but it influences mostly oral conditions such as ulcers [24], mucositis [25], and postoperative sore throat [26].

Our team already has experience in researching casein as a carrier of benzydamine [27]. In these studies, nanoparticles were synthesized using the nano-spray drying technique. Depending on the casein:Benz mass ratio, the encapsulation efficiency varied in a range from 34.6% to 78.8%. It was also shown that the release in artificial saliva during the first five hours of the experiment was between 70% and 95%. Initial burst release was observed at higher drug concentrations, which was probably due to the accumulation of benzydamine in the periphery of the nanoparticles during the spray-drying process.

In the present study, casein gels crosslinked with sodium tripolyphosphate have been loaded with benzydamine hydrochloride in order to obtain controlled release and to avoid the undesirable initial burst effect in the release process. The influence of the polymer concentration, polymer:crosslinker ratio, and the presence of ethanol as a cosolvent of the benzydamine hydrochloride were examined. The benzydamine carriers obtained in this way are planned to be used for the buccal route of administration.

## 2. Materials and Methods

### 2.1. Materials

Sodium tripolyphosphate and casein (CAS) (casein sodium salt from bovine milk) were delivered by Sigma Aldrich (St. Louis, MO, USA). Benzydamine hydrochloride (Benz) was purchased from Alfa Aesar (Thermo Fisher (Kandel) GmbH, Kandel, Germany). Ethanol and other solvents were used with analytical grades of purity. Ultra-pure water was purified with the system Adrona Crystal B30 Bio with conductivity of 0.055 µS/cm.

### 2.2. Sample Preparation

CAS gels were formed by ionotropic gelation in the presence of phosphate ions from the sodium tripolyphosphate (NaTPP) used as a crosslinking agent. Namely, 1% or 2% *w*/*v* CAS water solutions, adjusted to pH 3 by HCl, were prepared. In some cases, 5% *v*/*v* of ethanol used as a cosolvent was dropped into the solution. Then, 40 mg of Benz was added dropwise to this solution. Afterwards, it was left to stir for 30 min at room temperature before the addition of NaTPP. The crosslinker was added in a drop-wise manner in CAS:NaTPP ratios of 3:1, 5:1, and 10:1. This mixture was stirred for 2 h to react, and at the end of the reaction the suspensions were precipitated by centrifuging for 15 min at 14,000 rpm. Then, the precipitates from CAS particles were additionally washed with deionized water and centrifuged again for 10 min at the same speed. Finally, the samples were freeze-dried for 72 h and kept in vacuum containers for further use.

### 2.3. Characterization

#### 2.3.1. Dynamic Light Scattering (DLS)

The sizes of the casein structures were examined by the method of dynamic light scattering. The type of equipment used for these measurements was the Nanotrac particle size analyzer (Microtrac, York, PA, USA).

#### 2.3.2. Atomic Force Microscopy (AFM)

The shape, size, and aggregation phenomena of blank particles were investigated by atomic force microscopy (AFM) (Nanosurf FlexAFM (Nanosurf AG, Liestal, Switzerland)). The particles were redissolved in distilled water and the sample suspension was deposited on a freshly cleaned microscopic glass. One minute after the deposition, the surface was rinsed with distilled water. The sample was left to dry for 24 h. The images were collected in tapping mode of the AFM using standard cantilever Tap190Al-G (Nanosurf AG, Liestal, Switzerland) with a 10 nm tip radius. The resultant picture showed a 10 × 10 µm area from the sample surface with a viewing field of 256 × 256 pixels collected over a 0.7 s scan time.

#### 2.3.3. Scanning Electron Microscopy (SEM)

Scanning electron microscopy (SEM) (Prisma E SEM, Thermo Scientific, Waltham, MA, USA) was used to characterize the morphology of the blank particles. The samples were loaded on a copper sample holder and sputter-coated with carbon followed by gold using a vacuum evaporator (BH30, Prisma E SEM, Thermo Scientific, Waltham, MA, USA). The images were recorded at a 15 kV acceleration voltage at various magnifications using a DBS (back-scattered electrons) detector (Prisma E SEM, Thermo Scientific, Waltham, MA, USA).

#### 2.3.4. Attenuated Total Reflection Fourier-Transform Infrared Spectroscopy (ATR-FTIR)

The spectra of the neat materials, crosslinked CAS gels, and CAS gels with loaded Benz were collected with a Nicolet iS 10 FTIR spectrometer (Thermo Fisher Scientific, Pittsburgh, PA, USA) in ATR mode. The operating range of the equipment was 600 to 4000 cm^−1^ with a resolution of 4 nm and 64 scans. The obtained experimental data were analyzed with the OMNIC^®^ software package (Version 7.3, Thermo Electron Corporation, Madison, WI, USA).

#### 2.3.5. Yield of the Gel-Forming Process

After the washing and freeze-drying processes, the amount of CAS that formed into gel was evaluated. The yield of the gelation was calculated based on the formula:

(1) $$\mathrm{Yield}\;\left(\%\right)=\frac{\mathrm{dry}\;\mathrm{mass}\;\mathrm{of}\;\mathrm{the}\;\mathrm{particles}}{\mathrm{total}\;\mathrm{dry}\;\mathrm{mass}\;\mathrm{in}\;\mathrm{the}\;\mathrm{formulation}}\times 100$$

#### 2.3.6. Encapsulation Efficiency

The Benz content in the designed micro- and nano-particles was determined spectrophotometrically using a preliminary determined calibration curve. The particles (20 mg) were dispersed in 20 mL of 2% acetic acid solution and ultra-sonicated for 30 min until complete dissolution of the polymer and of the drug. After filtration (Chromafil Xtra RC (Macherey-Nagel, Dueren, Germany), 0.45 mm) and appropriate dilution, the Benz concentration was determined by the UV-VIS spectrophotometer, Metertech SP-8001(Metertech Inc., Nangang, Taipei, Taiwan), at a wavelength of 307 nm. The encapsulation efficiency (EE) was calculated based on the equation:

(2) $$\mathrm{EE}\;\left(\%\right)=\frac{\mathrm{loaded}\;\mathrm{Benzydamine}\;\mathrm{Hydrochloride}\;}{\mathrm{total}\;\mathrm{dry}\;\mathrm{mass}\;\mathrm{in}\;\mathrm{the}\;\mathrm{formulation}}\times 100$$

#### 2.3.7. Differential Scanning Calorimetry (DSC)

Thermal stability of blank and loaded particles together with the solid state of the compounds were examined with differential scanning calorimetry equipment DSC 204F1 Phoenix (Netzsch Gerätebau GmbH, Selb, Germany). It worked on the heat flux principle and it was cooled with an intra-cooler. The calibration of the instrument for both heat flow and temperature was carried out with an indium standard (T_m_ = 156.6 °C, ΔH_m_ = 28.5 J/g). All of the examined samples were sealed in identical aluminum pans and an empty pan was used as a reference. The heating rate was 10 °C/min and the measurements were performed under argon atmosphere.

#### 2.3.8. Drug-Release Simulation and Mathematical Modeling of the Process

Amounts, equivalent to 10 mg loaded Benz, from different models of the gels were used in a simulation of the release process kinetics. The desired amounts of particles were suspended in 1 mL of saliva buffer (pH = 6.8). This suspension was placed into a dialysis bag, which was poured into 25 mL of the same saliva buffer at 37 °C. Samples of 3 mL were taken at selected time intervals for spectroscopic analysis at 307 nm, and after taking out each sample, an equivalent amount of buffer was added back to the release medium. During the experiment, the release medium was continuously stirred at 50 rpm.

#### 2.3.9. Statistical Analysis

The results were statistically analyzed by calculating the standard deviations using MS Excel (version 2016, Microsoft Corporation, Redmond, WA, USA). TableCurve™ 2D (version 5.01, Sigma-Aldrich, St. Louis, MO, USA) was used in non-linear regression of drug release modeling.

## 3. Results and Discussion

### 3.1. Formulation and Characterization of Blank CAS Particles

**Figure 1:**
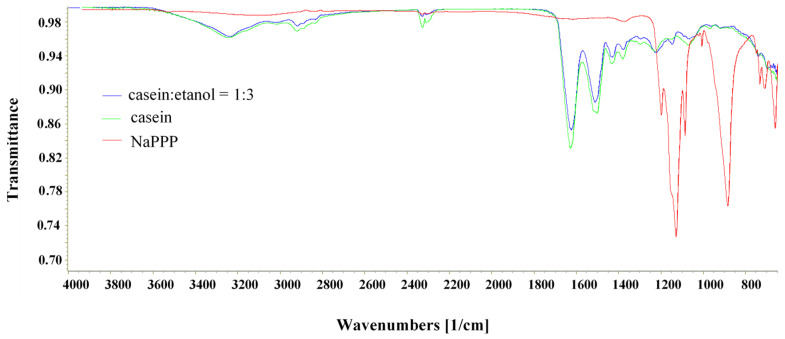
FTIR spectra of casein, sodium tripolyphosphate, and crosslinked casein particles.

Benz-loaded CAS particles were successfully prepared using ionic interactions for the crosslinking process. By decreasing the pH of the CAS solution below its isoelectric point, the amino groups in the CAS molecule were charged positive and could interact with the negatively charged phosphate groups in NaTPP, forming a crosslinked network. A similar mechanism of ionic crosslinking was reported already by Elzoghby [28]. The crosslinking process was confirmed by FTIR ATR analysis (Figure 1).

The spectra for casein showed strong amide I and amide II peaks between 1700 and 1500 cm^−1^ and the -NH group stretching at 3400–3000 cm^−1^ characteristic of amino acids, as it was previously reported by other authors [29]. Peaks at 1646 cm^−1^ in the amide I region and 1530 cm^−1^ in the amide II region could be assigned to the stretching of the carbonyl group (C=O) and to the symmetric stretching of N-C=O bonds, respectively [30]. Casein showed a band at 1077 and 977 cm^−1^, suggesting mono-cationic and di-cationic interactions with Na^+^ and Ca^2+^, respectively. In the spectrum of crosslinked casein, a new band at 1157 cm^−1^, which can be attributed to symmetric and antisymmetric stretching of PO_2_, was present and a shoulder appeared at 1210 cm^−1^, related to the antisymmetric stretching vibrations of PO_2_ groups of tripolyphosphate ions, which indicated the formation of ionic interactions between casein and NaTPP.

The casein particles exhibited a size in the range of 0.9 to 4.3 µm (Table 1). In general, these sizes were larger than the sizes of the particles obtained at neutral and alkaline pH and crosslinked with calcium [31]. Some of the samples were characterized with bimodal and trimodal distributions, which could be attributed to possible aggregation of the particles. The presence of ethanol led to increased aggregation and increased particle size. This effect was more pronounced at higher concentrations of casein [32].

**Figure 2:**
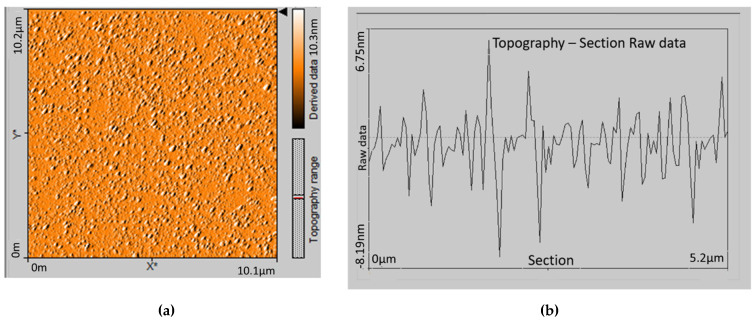
AFM image of empty casein particles (**a**) and cross-section (**b**). The sample was synthesized by 1% casein and casein:NaTPP ratio 3:1 without ethanol.

**Figure 3:**
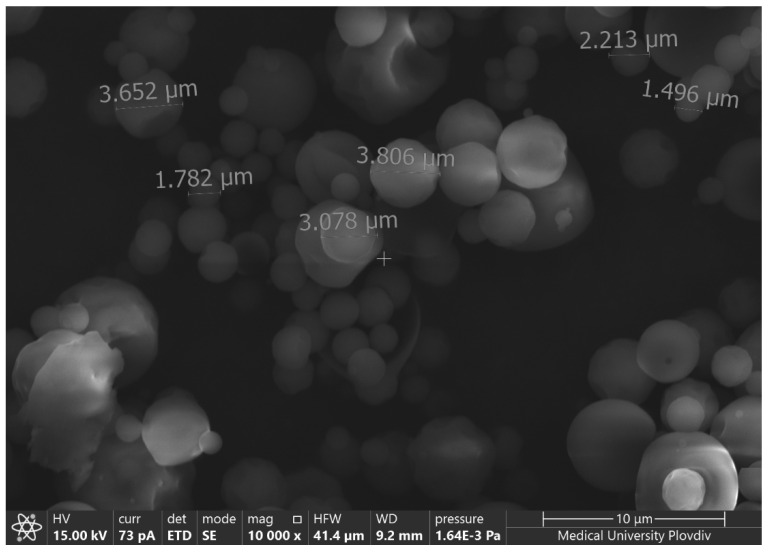
SEM microphotography of empty casein particles synthesized by 1% casein and casein:NaTPP ratio 3:1 without ethanol.

A couple of characteristics of the particles such as shape, size, and aggregation were examined with AFM and SEM techniques. A microphotograph of empty casein particles and their cross-section profile investigated by AFM is presented in Figure 2. SEM microphotographs of the same model are presented in Figure 3.

The AFM micrograph and SEM images demonstrated that the particle’s shape is not quite spherical, but closer to an irregular oval shape. No aggregation between the particles was detected. Similar results about casein micelles were reported by Ouanezar [33]. The sizes measured from the cross-section image confirm the results obtained by DLS.

A tendency for decreasing the particle size when the crosslinker concentration decreased was observed for the samples formulated from 2% casein concentration and in the presence of ethanol. Similar results were reported by other authors [28]. The acidic pH conditions under which casein particles were formed were suitable for the collapse of casein micelles. Thus, the particles obtained at higher micelle concentrations and low crosslinker concentrations were more unstable and collapsed to a greater extent. In all other tested samples, the decrease in crosslinker concentration led to an increase in size. This fact could be explained by the different crosslinking densities and the occurrence of a looser casein network. In that case, the particles could swell during the DLS measurements.

The yield of the gelation process was high for all investigated samples and varied between 55.3% and 78.3%. Practically, it did not depend on the casein concentration and the presence of ethanol. The highest yield was achieved for the samples with a NaTPP/casein ratio of 1:5.

### 3.2. Characterization of Benzydamine-Loaded Particles

#### 3.2.1. Drug Encapsulation Efficiency

Drug encapsulation efficiency varied in a wide range from 4.6% to 22.4% (Table 1). A tendency for a decrease in encapsulation efficiency was observed at the lowest concentration of the crosslinker. Probably, in this case, the structures were so loose and porous that it was difficult to entrap the low molecular weight drug. At first glance, it seemed surprising that the maximum encapsulation efficiency was not observed in the densest casein network prepared with the highest amount of crosslinking agent. The explanation could be that in this case, there is a certain collapse of the protein network, in which the hydrophilic drug was released during the synthesis of the particles. This hypothesis was also confirmed by the fact that a lower encapsulation efficiency was observed in systems produced in the presence of alcohol. Higher amounts of casein increased the encapsulation efficiency of benzydamine in the nanoparticles, which was probably due to the enhanced hydrophobic effect favoring micellar solubilization of the drug. Similar results were achieved by Elzoghby [34]. The addition of ethyl alcohol reduced the encapsulation efficiency of benzydamine, with the exception of the samples obtained with 1% casein and crosslinker 1:10, in which it helped to stabilize the structure. Therefore, further investigations on the benzydamine release behavior were performed with samples without added alcohol.

#### 3.2.2. Solid-State Characterization

**Figure 4:**
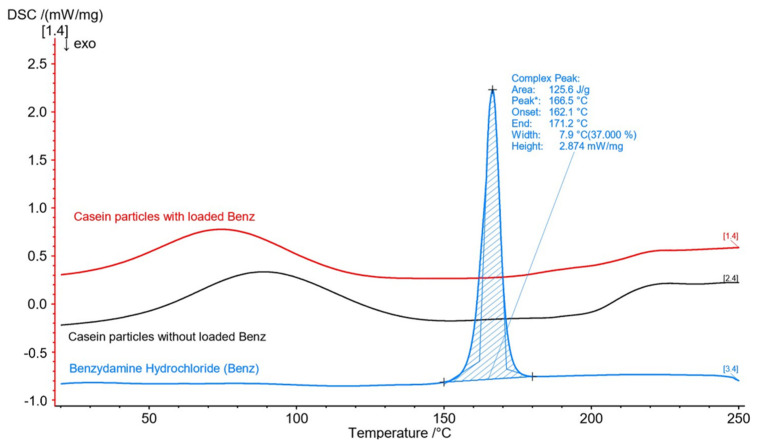
DSC thermogram of free benzydamine, casein particles without loaded drug, and casein particles with loaded benzydamine.

The differential scanning calorimetry (DSC) technique was applied in order to obtain the phase state of the bare compounds along with their thermal stability before and after the particles’ formation. The resulting thermograms are shown in Figure 4.

The casein particles’ thermogram showed a broad endothermic peak at about 90 °C, which was related to water evaporation from the samples, and another peak above 200 °C, which was due to thermal destruction. The free benzydamine was a crystal solid with a pronounced narrow endothermic peak corresponding to a melting temperature of 166.5 °C [35]. In the drug-loaded samples, no peak corresponding to benzydamine melting was observed. Therefore, an assumption could be made that loaded benzydamine existed in an amorphous state. The transformation from crystal to an amorphous solid most probably occurred when dissolving benzydamine in casein. These results were confirmed in all tested samples without any noticeable effect of casein or crosslinker concentration.

#### 3.2.3. In Vitro Benzydamine Release and Release Kinetics

**Figure 5:**
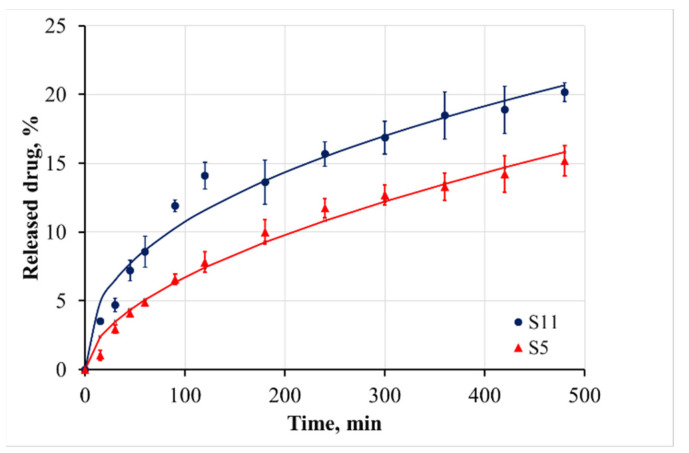
Cumulative release of benzydamine from casein particles, formulated at different casein concentrations.

**Figure 6:**
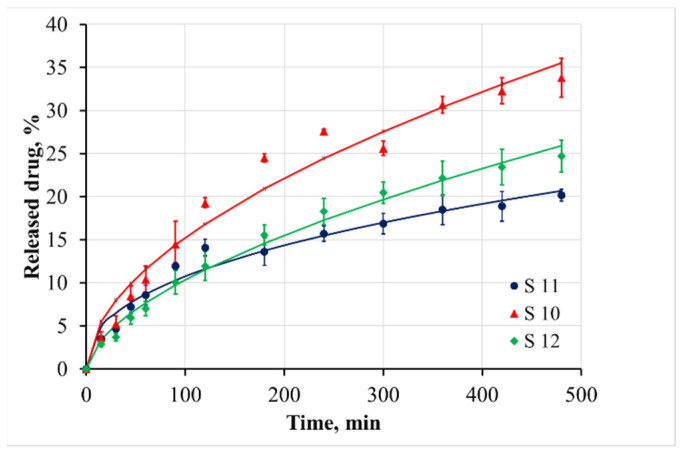
Cumulative release of benzydamine from casein particles, formulated at different crosslinker:casein ratios.

The cumulative release of benzydamine in PBS medium at pH 6.8 and 37.0 ± 0.5 °C from 0 to 480 min is shown in Figure 5 and Figure 6. The release during the first 8 h was incomplete, varying in the range between 15% and 32% depending on the sample composite. It demonstrated no burst effect of surface-adhered drug. Therefore, it could be concluded that the benzydamine was tightly entrapped in the bulk of casein micelles. This was the reason that the benzydamine release from the casein particles was slower in comparison to that from polysaccharide nanostructures, for example chitosan nanospheres [36]. Our hypothesis of the entrapment mechanism of benzydamine in the casein particles was confirmed by the fact that the increase of casein concentration led to a slower release (Figure 5). A similar tendency was observed when the crosslinker:casein ratio increased from 1:10 to 1:5 (Figure 6).

The denser network of the protein hindered the free diffusion of the drug and it moved slower from the inside of the particle to the surface.

When the crosslinker:casein ratio was 3:1, the initial release of the drug during the first 8 h was the fastest between the all investigated varieties. However, if we examined the dissolution of benzydamine after 72 h (Table 2), it was found that in the long run, this type of sample released slower. Therefore, it could be assumed that as a result of the denser micelle structure in the core of the particles, part of the benzydamine accumulated in the periphery. It was this less entrapped benzydamine that was initially released more rapidly, while the diffusion of benzydamine entrapped inside was very difficult.

To understand the processes responsible for the benzydamine release mechanism, the cumulative release data were fitted to the following models.

- First-order model, which can be used to describe the drug dissolution in pharmaceutical forms containing water-soluble drugs in porous matrices [37]:
  (3) $$\mathrm{logC}={\mathrm{logC}}_{0}-\frac{\mathrm{Kt}}{2.303}$$
  where C_0_ is the initial concentration of the drug, K is the first-order rate constant, and t is the time.
- Korsemeyer–Peppas model:
  (4) $$\frac{M_{t}}{M_{\infty }}={\mathrm{Kt}}^{n}$$
  where $\frac{M_{t}}{M_{\infty }}$ is the fraction of drug released at the time t, K is the Korsemeyer–Peppas constant, and the exponent n indicates the diffusion mechanism [38].
- Weibull model, which is useful for comparing the release profiles of matrix-type drug delivery [39]:
  (5) $$\frac{M_{t}}{M_{\infty }}=1-\exp\left[-\frac{{\left(t-T\right)}^{b}}{a}\right]$$
  where $\frac{M_{t}}{M_{\infty }}$ is the fraction of drug released at the time t, and T accounts for the lag time measured as a result of the dissolution process. Parameter a describes the time dependence, while b describes the shape of the dissolution curve progression. For b = 1, the shape of the curve corresponds exactly to the shape of an exponential profile; if b has a higher value than 1, the shape of the curve becomes sigmoidal with a turning point, whereas the shape of the curve with b lower than 1 would show a steeper increase than the one with b = 1.

The non-linear regression models were analyzed based on [40].

The values of the calculated parameters are presented in Table 3. In all experimental series, the T parameter in the Weibull model is 0 and therefore it is not shown in the table.

Based on the DF Adj r^2^ and F-values, it can be concluded that the kinetics of benzydamine release cannot be sufficiently accurately described with the first-order model. The Korsemeyer–Peppas model and the Weibull model could be applied to analyze the drug release. Based on the calculated n values from the Korsemeyer–Peppas model, it could be concluded that the release behavior of benzydamine out of the particles is governed by an anomalous (non-Fickian) diffusion mechanism. This mechanism includes the presence of both swelling-controlled and diffusion-controlled release. According to the literature, the casein micelles are stable at neutral pH [41], and therefore the benzydamine release in the samples is predominantly due to diffusion from the core to the periphery of the particles. The slowest release from sample S5—particles prepared from a higher casein concentration—was confirmed by the smallest value of the K parameter in the Korsemeyer–Peppas model and the highest value of the parameter a in the Weibull model. The lowest value of the parameter b for the sample S11 demonstrated the steepest increase of the benzydamine release.

## 4. Conclusions

Benzydamine-loaded casein micro-particles were produced by an ionotropic gelation mechanism. The particle sizes varied between 0.9 and 4.3 µm. The loading efficiency depended on the polymer/crosslinker ratio and increased with increasing the NaTPP concentration. During the entrapment, the benzydamine changed its phase state from crystal to amorphous. The drug release study confirmed our hypothesis that the casein particles were suitable to be used as carriers for prolonged benzydamine delivery, governed by a non-Fickian diffusion mechanism.

## Figures and Tables

**Table 1:** Size, yield, and encapsulation efficiency (EE) of casein particles.

Sample	CAS Concentration, % *w*/*v*	CAS:NaTPP Ratio	Ethanol Concentration, % *v*/*v*	Size, μm	Yield, %	EE, %
S1	2	3:1	5	3.9 ± 0.1 ^1^	64.4 ± 3.4	14.7 ± 0.8
S2	2	5:1	5	3.2 ± 0.1 ^1^	75.7 ± 4.8	22.2 ± 1.2
S3	2	10:1	5	2.2 ± 0.1 ^1^	68.9 ± 4.0	9.9 ± 0.5
S4	2	3:1	0	2.0 ± 0.1	66.6 ± 4.9	19.2 ± 1.0
S5	2	5:1	0	0.9 ± 0.0 ^2^	73.8 ± 4.3	22.4 ± 1.3
S5	2	10:1	0	2.4 ± 0.1	55.3 ± 3.6	5.4 ± 0.3
S7	1	3:1	5	2.5 ± 0.1 ^1^	65.2 ± 4.6	8.0 ± 0.5
S8	1	5:1	5	1.6 ± 0.0 ^2^	76.1 ± 4.3	13.7 ± 0.8
S9	1	10:1	5	4.3 ± 0.1	72.5 ± 4.2	4.6 ± 0.3
S10	1	3:1	0	1.2 ± 0.0	63.9 ± 4.6	18.2 ± 0.9
S11	1	5:1	0	2.1 ± 0.1 ^1^	71.4 ± 4.4	12.5 ± 0.7
S12	1	10:1	0	2.2 ± 0.1	78.3 ± 4.9	10.6 ± 0.6

^1^ Bimodal distribution is observed. ^2^ Trimodal distribution is observed.

**Table 2:** Released amounts of benzydamine until 72 h.

Sample	Released Amount (%) after:		
	24 h	48 h	72 h
S10	51.2 ± 3.9	55.4 ± 3.1	85.9 ± 6.7
S11	39.3 ± 4.3	63.2 ± 4.3	90.1 ± 9.0
S12	44.5 ± 2.6	62.2 ± 4.0	94.6 ± 3.8
S5	31.7 ± 3.5	48.3 ± 3.6	84.4 ± 5.1

**Table 3:** Model parameters of the first-order model, the Korsemeyer–Peppas model, and the Weibull model.

Sample	Model Parameters		DF Adj r^2^	Fit SE	F-Value
	**First-Order Model**				
	logC_0_	K			
S10	1.974 ± 0.008	(80 ± 8) × 10^−5^	0.898	0.02	118.3
S11	1.976 ± 0.005	(40 ± 5) × 10^−5^	0.823	0.01	63.8
S12	1.984 ± 0.004	(58 ± 4) × 10^−5^	0.942	0.01	217.5
S5	1.988 ± 0.003	(33 ± 3) × 10^−5^	0.900	0.01	130.0
	**Korsemeyer–Peppas Model**				
	K	n			
S10	0.013 ± 0.003	0.53 ± 0.04	0.963	2.1	352.5
S11	0.016 ± 0.003	0.51 ± 0.03	0.964	1.1	356.6
S12	0.007 ± 0.001	0.58 ± 0.02	0.989	0.8	1237.1
S5	0.005 ± 0.001	0.54 ± 0.03	0.983	0.7	754.7
	**Weibull Model**				
	b	a			
S10	0.61 ± 0.04	100 ± 25	0.969	2.0	422.1
S11	0.45 ± 0.03	69 ± 13	0.967	1.1	383.7
S12	0.63 ± 0.02	172 ± 23	0.990	0.7	1663.8
S5	0.57 ± 0.03	200 ± 33	0.980	0.6	853.0

## Data Availability

The data presented in this study are available upon request from the corresponding author.
